# Impact of the Body Mass on Complications and Outcome in Multiple Trauma Patients: What Does the Weight Weigh?

**DOI:** 10.1155/2013/345702

**Published:** 2013-08-19

**Authors:** Hagen Andruszkow, Juliane Veh, Philipp Mommsen, Christian Zeckey, Frank Hildebrand, Michael Frink

**Affiliations:** ^1^Department of Trauma and Reconstructive Surgery, University Hospital Aachen, Pauwelsstraße 30, 52074 Aachen, Germany; ^2^Trauma Department, Hannover Medical School, Carl-Neuberg-Street 1, 30625 Hannover, Germany; ^3^Department for Trauma, Hand and Reconstructive Surgery, University Medical Center Marburg, Baldingerstraße, 35043 Marburg, Germany

## Abstract

Obesity is known as an independent risk factor for various morbidities. The influence of an increased body mass index (BMI) on morbidity and mortality in critically injured patients has been investigated with conflicting results. To verify the impact of weight disorders in multiple traumatized patients, 586 patients with an injury severity score >16 points treated at a level I trauma center between 2005 and 2011 were differentiated according to the BMI and analyzed regarding morbidity and outcome. Plasma levels of interleukin- (IL-) 6 and C-reactive protein (CRP) were measured during clinical course to evaluate the inflammatory response to the “double hit” of weight disorders and multiple trauma. In brief, obesity was the highest risk factor for development of a multiple organ dysfunction syndrome (MODS) (OR 4.209, 95%-CI 1.515–11.692) besides injury severity (OR 1.054, 95%-CI 1.020–1.089) and APACHE II score (OR 1.059, 95%-CI 1.001–1.121). In obese patients as compared to those with overweight, normal weight, and underweight, the highest levels of CRP were continuously present while increased systemic IL-6 levels were found until day 4. In conclusion, an altered posttraumatic inflammatory response in obese patients seems to determine the risk for multiple organ failure after severe trauma.

## 1. Introduction

Obesity and overweight represent risk factors for chronic diseases emphasizing diabetes mellitus, hyperlipidemia, heart disease, hypertension, and so forth [1]. While in the United States actually two thirds of the adult population are overweight or obese, obesity is continuously increasing in European citizens with currently approximately 20% [1, 2]. In contrast, the incidences of eating disorders resulting in significant underweight are increasing as well, revealing one of the most common health problems in female adolescents and young women [3, 4]. Physical complications are common among these patients based on endocrinological, electrolyte, hematological, and metabolic abnormalities [3, 4]. Consequently, obesity as well as underweight induce anatomical and physiological changes interfering with the body's response to trauma and emergency surgery [5]. However, consequences of weight disorders on complications and outcome following trauma and emergency surgery are still poorly understood and conflicting results have been reported [6, 7]. Focusing on the relationship between body weight and mortality, a U-shaped correlation between the body mass index and in-hospital mortality is described [2, 8] demonstrating increased mortality in underweight but less markedly in obese patients [2, 7, 8]. Nevertheless, increased morbidity is found in obese patients manifested in impaired hemodynamics and tissue perfusion leading to an increased incidence of multiple organ failure after elective surgery [9]. Consequently, following the literature on critical care medicine, increased mortality in obese patients emphasizing on critical airway management, difficult surgical exposures, challenging nursing care, and complicated diagnostics is expected [5, 10, 11]. Emerging theories are currently suggesting that trauma- and obesity-induced inflammatory stress plays a pivotal role in increased morbidity and mortality in severe trauma combined with weight disorders [12, 13]. In general, trauma induces an immunological response with a complex acute-phase protein and cytokine release resulting in endothelial cell damage, dysfunction of vascular permeability, microcirculatory disturbances, and necrosis of parenchymal cells [14]. Among inflammatory markers, Interleukin- (IL-) 6 levels currently represent the best correlation with severity of injury and the risk of organ dysfunction [15–18]. In addition, several studies have elucidated increased IL-6 levels in obese patients correlated with insulin resistance [19, 20] and the incidence of diabetes [21]. 

The present study intends to clarify the relationship between body mass index, complications and outcome after severe multiple trauma. In order to understand the biochemical coherences in these patients in vivo, measurements of relevant inflammatory mediators are ascertained. 

## 2. Material and Methods

The present study follows the guidelines of the revised UN declaration of Helsinki in 1975 and its latest amendment in 1996 (42nd general meeting). 

### 2.1. Study Design and Inclusion Criteria

A retrospective analysis of all multiple traumatized patients, defined as Injury Severity Score (ISS) ≥16 points, primarily admitted to a level I trauma center between January 1, 2005 and June 30, 2011, was performed. Further inclusion criteria were admission to intensive care unit (ICU) within the first 24 hours and age ≥16 years. Patients with incomplete data referring to weight or height preventing BMI measurements were excluded.

### 2.2. Body Mass Index (BMI)

Admission weight and height were used to calculate the body mass index (BMI (kg/m^2^)). Patients were assigned to four groups based on the BMI using the classification of the World Health Organization as follows: Group I (underweight) (i) BMI < 20.0 kg/m^2^; Group II (normal weight) (ii) BMI 20.0–24.9 kg/m^2^; Group III (overweight) (iii) BMI 25.0–29.9 kg/m^2^; Group IV (obesity) (iv) BMI > 30.0 kg/m^2^. Demographics, mechanism of injury, injury distribution and severity, clinical course, serum markers, complications, and outcome were analyzed.

### 2.3. Injury Severity and Clinical Course

Injury distribution was determined with the 2005 revised edition of the Abbreviated Injury Scale (AIS) and summarized to the Injury Severity Score (ISS) reflecting the overall injury severity [22]. Each injury is assigned an AIS score according to its relative importance on a six-point scale (1, minor; 2, moderate; 3, serious; 4, severe; 5, critical; 6, unsurvivable) and is allocated to one of six body regions: head and neck; face; thorax; abdomen; extremities (including pelvis); and external. Only the highest AIS score in each body region is used. The three most severely injured body regions have their score squared and added together to the ISS [22].

Clinical course included duration of ventilation (hours), duration of ICU treatment (days), and the overall length of stay (LOS) in days. The duration of initial emergency surgery (minutes) was defined as any surgery performed in the operating room within the first 24 hours after hospital admission.

### 2.4. Complications and Outcome

In order to estimate the risk of mortality, the “Acute Physiology and Chronic Health Evaluation II” (APACHE II) score [23] and its predicted mortality were documented on admission to ICU after emergency surgery was performed. 

Complications analyzed included the “systemic inflammatory response syndrome” (SIRS), sepsis, the acute respiratory distress syndrome (ARDS), and the multiple organ dysfunction syndrome (MODS). SIRS and sepsis were classified according to the definitions for sepsis and organ failure [24]. ARDS was assumed following the recommendations of the “The American-European Consensus Conference on ARDS” [25, 26]. The incidence of MODS was categorized as described by Marshall et al. [27] considering multiple organ failure if the score was greater than 12 points on two consecutive days or at least three days during a 14-day observation period [27, 28]. primary outcome was defined as mortality during clinical course.

### 2.5. Inflammatory Biomarkers

C-Reactive protein (CRP) and IL-6 levels were measured with blood samples taken during emergency room management after hospital admission as a standard procedure. The samples represent the immunological response to trauma before emergency surgery was initiated. During the ICU stay, blood samples were taken repetitively every morning at 07:00 a.m. for at least 14 days after admission to ICU. 

### 2.6. Statistical Methods

The data were analyzed using the Statistical Package for the Social Sciences (SPSS; version 19; IBM Inc., Somers, NY, USA). Incidences are presented with counts and percentages while continuous values are presented as mean ± standard deviation (SD). Differences between the groups were evaluated with analysis of variance (ANOVA) for continuous data, while Pearson's *χ* ^2^-test was used for categorical values. A pairwise comparison was not performed due to an increased type I error. In order to reveal the impact of BMI on complications, a multivariate logistic regression analysis was performed with MODS as a target variable and BMI group, injury severity (ISS), and APACHE II score as potential predictors. Odds ratios with 95% confidence intervals (95%-CI) were noted. The Spearman rank correlation coefficient was used to determine the connection between IL-6 and CRP levels and development of MODS. A two-sided *P* value < 0.05 was considered to be significant.

## 3. Results

660 adult multiple traumatized patients were treated between January 1, 2005, and June 30, 2011. A total of 74 patients (11.2%) were excluded due to missing body weight or height data preventing BMI measurements. 

Including 586 patients, the overall mean BMI was 26.0 ± 4.5 kg/m^2^ (range 15.1–56.8 kg/m^2^). The male to female ration was 2.3 : 1 with a mean age of 42.0 ± 18.0 years.

### 3.1. BMI, Injury Severity, and Clinical Course

Diversifying the included trauma collective, 4.8% patients were underweight, 45.2% had normal weight, 36.0% were overweight, and 14.0% were obese. We elucidated significant differences between these four groups with regard to age, gender, and severity of head injuries (Table 1). Obese patients demonstrated the highest age and ratio of males while underweight patients were younger females (Table 1). Analyzing injury distribution, the lowest severity of head injuries was noted in obese patients while no differences were found regarding the overall injury severity as well as the remaining injury distribution (Table 1). 

Duration of ventilation, length of ICU treatment and the overall length of stay increased consistently with the BMI. Duration of initial emergency surgery was not influenced by the BMI at admission (Table 2).

### 3.2. Complications and Outcome

According to the measured APACHE II score and its predicted mortality, no differences could be demonstrated between the BMI groups (Table 3). Furthermore, no statistical differences referring to incidence of SIRS, sepsis, and ARDS were evaluated between the BMI groups. However, a strong trend towards an increased rate of MODS in obese patients was proven (Table 4). 

In a multivariate analysis, obesity was revealed as the highest risk factor for development of multiple organ dysfunction syndrome (OR 4.209, 95%-CI 1.515–11.692; *P* = 0.006) beside injury severity (OR 1.054, 95%-CI 1.020–1.089; *P* = 0.001) and the prognostic APACHE II score (OR 1.059, 95%-CI 1.001–1.121; *P* = 0.047) (Table 5). 

Regarding outcome, there was no influence of the nutrition status on mortality although mortality was increased in underweight patients without reaching statistical significance (*P* = 0.484). 

### 3.3. Inflammatory Biomarkers

**Figure 1:**
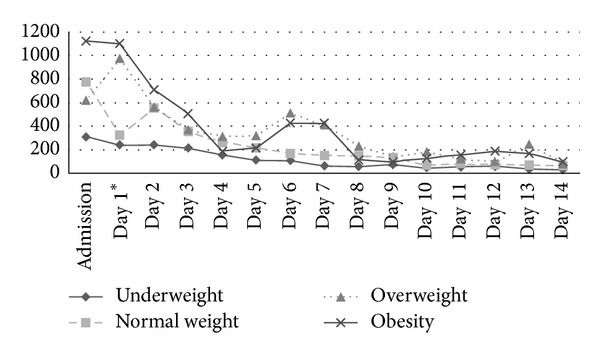
Mean IL-6 courses (pg/mL) evaluated from admission to day 14 according to the BMI groups. Significant differences (*P* < 0.05) are marked with an asterisk referring to the day of distinction.

**Figure 2:**
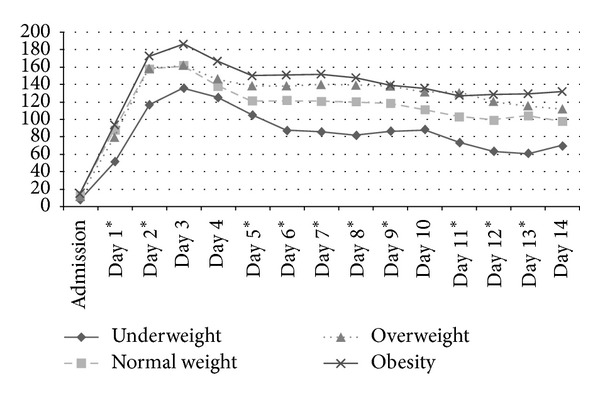
Mean CRP courses (mg/L) evaluated from admission to day 14 according to the BMI groups. Significant differences (*P* < 0.05) are marked with an asterisk referring to the day of distinction.

The descriptive course of IL-6 and CRP according to the different BMI groups is illustrated in Figures 1 and 2. Descriptively, obese patients have been admitted with increased mean plasma IL-6 levels (1,122.6 ± 4,376.5 pg/mL) followed by patients with normal weight (779.9 ± 1,741.6 pg/mL), overweight (620.9 ± 1,330.0 pg/mL), and underweight (310.0 ± 494.5 pg/ mL) (*P* = 0.470) (Figure 1). However, on day 1 IL-6 values in obese patients were comparable to levels found at admission while in overweight patients an increase was revealed. In contrast, IL-6 levels in normal and underweight patients were decreased at that time (*P* = 0.008). Overall, underweight patients demonstrated the lowest systemic IL-6 values during the observed period (Figure 1). Nevertheless, statistical significant differences of IL-6 levels according to the different BMI groups were only verified on day 1 (*P* = 0.008). Emphasizing on the impact of IL-6, a positive correlation with the incidence of MODS was present at any time during the study period (Table 6).

Regarding CRP values, obese patients demonstrated increased levels after admission without reaching statistical significance (BMI group I 7.5 ± 11.8 mg/L; BMI group II 12.6 ± 24.5 mg/L; BMI group III 11.5 ± 25.1 mg/L; BMI group IV 15.3 ± 25.6 mg/L; *P* = 0.452). A continuous increase with a maximum on day 3 was observed followed by a continuous decrease during the observation period. During the first 14 days highest levels were observed in obese patients followed by overweight, normal weight, and underweight patients. Significant differences between the illustrated CRP values according to the BMI groups were found from day 1 to day 3, from day 4 to 9, and day 11 to day 13 (Figure 2). Increased CRP values were positively correlated with the incidence of MODS from day 3 (Table 6).

## 4. Discussion

The influence of an increased body mass index on morbidity and mortality in critically injured patients has shown conflicting results [5, 29, 30]. However, the present study provides new insights regarding impact of body weight in multiple traumatized patients evaluating the posttraumatic systemic immune response. The main results of the present study could be summarized as follows:

- obesity was revealed as highest risk factor for development of MODS while no influence on mortality was shown. Although not reaching statistical significance, underweight patients showed twice the mortality rate as compared to obese patients which may be of clinical significance and should be considered in future diagnostic and therapeutic steps,
- obese patients have been admitted with increased IL-6 levels for the first four days. Overall, underweight patients demonstrated the lowest IL-6 values during the observed period,
- measuring systemic CRP levels, obese patients had significantly increased values for almost the complete study period,
- increased IL-6 levels were positively correlated with the incidence of MODS during the whole study period, while increased CRP levels were positively correlated with MODS after day 3.

The first study investigating the relationship between body mass and trauma was performed in 1991 by Choban et al. [11] including 184 multiple traumatized patients. The authors showed a 42% mortality rate in obese patients compared to 5% in those with normal weight. Due to the remaining question, if this tremendous difference in mortality is based upon a specific injury pattern, Boulanger et al. focused on the body habitus as a predictor for injury pattern [10]. Interestingly, obese trauma victims were more likely to suffer from severe thoracic injuries but incidence of severe head injuries decreased [10]. According to the results of our study with less severe head injuries in obese patients, obesity seems to influence the injury pattern even 20 years later in the same way despite improving active and passive vehicle safety systems [31]. 

Several reports were recently performed due to increasing public awareness of the obesity epidemic and its wide influence [30]. Referring to differences in study designs diverse findings according to morbidity and mortality in obese patients were revealed [2, 5–9, 29]. In the current study we were able to identify obesity as the most important risk factor for the development of MODS but interestingly without significantly influencing mortality. In line with these results, obesity has been verified as an isolated risk factor for postinjury multiple organ failure with an odds ratio of 1.8 beside age, massive blood transfusion, and injury severity in a recent study [29]. Moreover, obesity increased length of ICU treatment and in-hospital time [29]. Similar to the presented results no differences were evaluated referring to mortality. The prolonged requirement of ICU treatment and in-hospital time in our as well as other studies may be a direct consequence of increased incidence of MODS [32, 33]. According to Newell et al., obesity in traumatized patients influenced multiple organ failure with a risk of 2.6 odds ratio without effecting mortality (OR 0.81) [30]. In contrast, Neville and colleagues showed an increased incidences of multiple organ failure (13% versus 3%) and increased mortality rates (32% versus 16%) in obese critically injured patients [5]. Contrary to the presented results, the authors assigned their patients only to two groups, obese (BMI ≥ 30) and nonobese (BMI < 30) which might not adequately consider the complexity of weight disorders. One of the largest studies with data of 5,766 traumatized patients from the Trauma Registry of the German Trauma Society showed an increased incidence of multiple organ failure in obese patients while underweight was associated with a lower incidence [7]. Similar to our results no differences between the BMI groups were revealed referring to mortality in a direct comparison. But according to a multivariate analysis, underweight as well as obesity were associated with increased mortality [7]. In brief summary, considering these studies an indisputable association of weight disorders and morbidity and respectively mortality was shown. Although these results are confirmed by data of the present study, to date no potential reasons for the increased morbidity and mortality were proven. One explanatory approach was found by Belzberg et al., who measured reduced tissue perfusion and cardiac output in obese nonsurvivors following severe trauma [9]. Consequently, emerging theories suggested that the cytokine response to trauma as well as surgical interventions might be altered in obesity. Lately, IL-6 as a part of the proinflammatory cascade was verified as the most reliable prognostic marker following trauma [16]. IL-6 not only correlates with injury severity [17] but is highly associated with multiple organ failure and outcome [16, 18]. Congruent to these findings, increased levels of IL-6 were strongly correlated with the incidence of MODS during the whole study period in the presented study. However, increased systemic concentrations of proinflammatory cytokines such as IL-6 are associated with systemic insulin resistance [19, 20] and the incidence of diabetes [21]. Considering that basal systemic IL-6 concentrations are enhanced in obese patients increased plasma levels could be expected following major trauma. Following surgical stress, Gletsu et al. monitored plasma and adipose tissue concentrations of IL-6 after abdominal surgery [13]. The circulating IL-6 concentrations at baseline and after surgery were related to the abdominal adipose tissue content and exaggerated in obese patients. After surgery, worsening of insulin resistance was correlated with increased systemic as well as adipose tissue content of IL-6 [13]. The effect of surgical interventions on IL-6 release might be similar in major trauma considering the presented results. Comparing systemic IL-6 levels on admission and day one, considerable differences of IL-6 values dependent on the nutrition status were found demonstrating a systemic increase in overweight and obese patients as compared to normal-weight and underweight patients. Since the durations of the initial surgery and the injury severity were comparable between BMI groups, an influence of the nutrition status could be suggested. An increased initial inflammatory state illustrated by systemic IL-6 levels was shown to enhance the sensitivity and vulnerability to trauma [34]. Thus, elevated systemic IL-6 levels in obese trauma victims on admission in the presented study may be from relevance for the further clinical course. In addition, the lowest levels of IL-6 in underweight may result of reduced adipose tissue and fatty acids which release inflammation-related adipokines commonly related to obesity-associated pathologies [35]. Contrary to the proinflammatory adipokines, adiponectin acts as insulin-sensitizing, antiatheroganic hormone with an anti-inflammatory potential [36]. This interaction of pro- and anti-inflammatory mediators is demonstrated to be negatively influenced in obesity which has been characterized as a continuous state of mild inflammation [35, 37, 38]. 

Measuring an inflammatory status, the C-reactive protein has been proven to be one of the most sensitive inflammatory markers [37, 38]. Furthermore, current studies revealed an association with atherosclerotic diseases and diabetes mellitus type II [39, 40]. Within the regulation of the inflammatory cascade, CRP suppresses adiponectin exaggerating to the inflammatory reaction in obesity [37, 38]. This exaggeration of inflammation could be demonstrated according to the presented results illustrating increased systemic CRP levels for almost the complete study period in obese patients. Similar to our suggestions referring the IL-6 values, the highest measurements of CRP in obese patients and, respectively, the lowest in underweight seemed not to be influenced by injury severity, duration of emergency surgery, or the incidences of clinical complications. Similarly, Kraft et al. demonstrated increased CRP and triglyceride levels in overweight pediatric burn patients [41]. The authors explained this finding with the increased proinflammatory, catabolic state of obese patients [41]. Although the prognostic power of CRP referring to complications is debatable [42, 43], increased CRP levels were correlated with the incidence of MODS after day 3 in the present study. 

However, the presented study has notable limitations. Apriori, a substantial number of patients had to be excluded due to missing weight and height data preventing the BMI measurement. This limiting aspect is known in comparable studies [2, 5, 7, 8, 30], wherein missing data lead to exclusion up to 17%. In addition, excluded patients in the presented study had the same characteristics as compared to the whole population (data not shown). Therefore, we do not expect this subgroup to compromise the demonstrated results. Nevertheless, one can argue whether the BMI represents an accurate tool to determine weight disorders. Even though a correlation exists between BMI and body fat (%), classification of individual weight disorders may show discrepancies when body fat analyses were performed [44]. Although divergent results have been demonstrated whether BMI or body fat measurements are more accurate to determine potential outcome prognoses [45], we feel safe to use BMI measurement since it represents the mostly accepted parameter in the current literature analyzing trauma populations [2, 5, 7, 8, 30, 41]. Emphasizing our suggestions of adipokines and adiponectin influencing the inflammatory response in multiple trauma, this aspect could not be proven due to the retrospective design of this study. These hormones are not measured as standard laboratory parameters in our multiple trauma patients. To field this limiting aspect, further research is required in order to verify presumable correlations between IL-6 and CRP with these hormones, which have been related to common obesity-associated pathologies.

## 5. Conclusion

According to the presented results, obesity was revealed as an independent risk factor for the development of MODS in severely traumatized patients. In obese patients, systemic IL-6 levels were elevated until day four while CRP presented the highest levels in obese patients followed by overweight, normal-weight, and underweight patients during the whole period. In conclusion, an altered inflammatory reaction following the “double hit” of obesity and multiple trauma seems to determine the risk for multiple organ failure after severe trauma. Thus, the nutrition status seems to play a pivotal role in the posttraumatic clinical course and therefore should be considered in therapeutic strategies in patients suffering from major trauma. 

Further research with attention to obese patients might ascertain presumable correlations of IL-6 and CRP with adipokines as well as adiponectin, which are commonly related to obesity-associated pathologies. 

## Figures and Tables

**Table 1:** Study population and characteristics according to the BMI.

	All	Underweight	Normal	Overweight	Obesity	*P* value
Number of patients (%)	586 (100.0%)	28 (4.8%)	265 (45.2%)	211 (36.0%)	82 (14.0%)	—
Age (years)	**42.0 ± 18.0**	**33.6 ± 19.7**	**37.2 ± 17.4**	**46.7 ± 17.7**	**48.0 ± 14.9**	**<0.001**
Male (%)	**410 (70.0%)**	**9 (32.1%)**	**176 (66.4%)**	**162 (76.8%)**	**63 (76.8%)**	**<0.001**
GCS	10.5 ± 4.8	9.5 ± 5.1	10.1 ± 4.8	10.8 ± 4.7	11.4 ± 4.7	0.066
AIS head	**1.9 ± 1.8**	**2.4 ± 2.0**	**2.0 ± 1.8**	**1.9 ± 1.8**	**1.5 ± 1.5**	**0.034**
AIS face	1.0 ± 1.2	1.0 ± 1.2	1.0 ± 1.3	1.0 ± 1.2	0.8 ± 1.0	0.337
AIS chest	2.7 ± 1.5	2.6 ± 1.5	2.6 ± 1.5	2.7 ± 1.6	3.0 ± 1.2	0.175
AIS abdomen	1.2 ± 1.6	1.5 ± 1.8	1.2 ± 1.6	1.1 ± 1.5	1.3 ± 1.6	0.485
AIS extremities	2.1 ± 1.4	1.8 ± 1.4	2.2 ± 1.4	2.1 ± 1.4	2.2 ± 1.3	0.285
AIS extern	1.0 ± 1.1	1.0 ± 1.0	1.1 ± 1.1	1.0 ± 1.1	1.1 ± 1.1	0.822
ISS	28.8 ± 10.7	29.8 ± 11.2	29.1 ± 10.7	28.9 ± 11.1	27.1 ± 9.7	0.502

GCS: Glasgow Coma Scale.

AIS: Abbreviated Injury Scale.

ISS: Injury Severity Score.

**Table 2:** Clinical course according to the BMI.

	Underweight	Normal	Overweight	Obesity	*P* value
Duration of initial emergency surgery (min.)	94.6 ± 81.7	85.0 ± 87.3	90.1 ± 89.5	105.4 ± 87.7	0.329
Duration of ventilation (hours)	191.1 ± 211.0	258.0 ± 304.1	329.7 ± 403.7	359.3 ± 366.9	0.028
Duration of ICU treatment (days)	11.6 ± 8.9	14.3 ± 13.7	17.4 ± 18.0	18.6 ± 17.5	0.044
Length of stay (days)	20.2 ± 10.5	25.7 ± 18.8	28.4 ± 22.6	34.4 ± 26.0	0.005

**Table 3:** Risk profile measured by the APACHE II score, its expected mortality, and outcome.

	Underweight	Normal	Overweight	Obesity	*P* value
APACHE II (points)	14.2 ± 6.8	12.7 ± 6.9	13.9 ± 7.4	14.1 ± 7.0	0.228
Expected mortality (%)	23.0 ± 15.0	20.0 ± 14.4	23.0 ± 16.8	22.7 ± 16.6	0.192
Observed mortality (%)	5 (17.9%)	25 (9.4%)	24 (11.4%)	7 (8.5%)	0.484

**Table 4:** Clinical complications according to the BMI.

	Underweight	Normal	Overweight	Obesity	*P* value
SIRS	16 (57.1%)	189 (71.3%)	156 (73.9%)	62 (75.6%)	0.256
Sepsis	9 (32.1%)	107 (40.4%)	91 (43.1%)	37 (45.1%)	0.612
ARDS	10 (35.7%)	78 (29.4%)	72 (34.1%)	31 (37.8%)	0.463
MODS	1 (3.6%)	9 (3.4%)	12 (5.7%)	9 (11.0%)	0.060

SIRS: Systemic Inflammatory Response Syndrome.

ARDS: Acute Respiratory Distress Syndrome.

MODS: Multiple Organ Dysfunction Syndrome.

**Table 5:** Multivariate regression analysis referring to MODS analyzing BMI, injury severity (ISS), and APACHE II score as potential predictors.

Predictor	Regression coefficient	Odds ratio (OR)	95% confidence interval (95%-CI)	*P* value
Underweight	0.254	1.289	0.152–10.9666	0.816
Overweight	0.606	1.832	0.718–4.675	0.205
Obesity	**1.437**	**4.209**	**1.515–11.692**	**0.006**
Injury severity (ISS)	**0.053**	**1.054**	**1.020–1.089**	**0.001**
APACHE II	**0.058**	**1.059**	**1.001–1.121**	**0.047**
Constant	−5.935	—	—	<0.001

BMI group II (normal weight) was set as a categorical reference group for regression analysis between the BMI groups.

**Table 6:** Correlation of systemic plasma IL-6 and CRP values with the incidence of MODS during the clinical course.

	Correlation coefficient IL-6	Correlation coefficient CRP
Admission	0.222*	0.054
Day 1	0.141*	0.045
Day 2	0.154*	0.036
Day 3	0.148*	0.093*
Day 4	0.170*	0.120*
Day 5	0.122*	0.130*
Day 6	0.241*	0.129*
Day 7	0.186*	0.122*
Day 8	0.175*	0.129*
Day 9	0.154*	0.156*
Day 10	0.184*	0.183*
Day 11	0.266*	0.191*
Day 12	0.250*	0.239*
Day 13	0.261*	0.271*
Day 14	0.197*	0.277*

Spearman's rank correlation; *P < 0.05.
